# The effects of moderate intensity training in a hypoxic environment on transcriptional responses in Thoroughbred horses

**DOI:** 10.1242/bio.020388

**Published:** 2017-06-05

**Authors:** Allan J. Davie, Li Wen, Andrew R. E. Cust, Rosalind Beavers, Tom Fyfe, Shi Zhou

**Affiliations:** 1 Southern Cross University, School of Health and Human Sciences, Lismore, NSW 2480, Australia; 2 Tianjin University of Sports, Tianjin Key Laboratory of Exercise Physiology and Sports Medicine, Tianjin 300381, China; 3 Ballarat Veterinary Practice, Ballarat, VIC 3350, Australia; 4 Pulford Air and Gas, Sydney, NSW 2141, Australia

**Keywords:** Hypoxia, Thoroughbred, Training, Muscle, Gene expression

## Abstract

This study investigated the effects of six weeks of normobaric hypoxic training on transcriptional expression of the genes associated with mitochondrial and glycolytic activities in Thoroughbred horses. Eight horses were divided into two groups of four. They completed an identical incremental, moderate intensity training program, except that one group trained in a hypoxic chamber with 15% oxygen for 30 min on alternate days except Sundays (HT), while the other group trained in normal air (NC). Prior to and post training, heart rate and blood lactate were measured during an incremental treadmill test. Muscle biopsy samples were taken prior to and 24 h post the training period for qPCR analysis of mRNA changes in VEGF, PPARγ, HIF-1α, PGC-1α, COX4, AK3, LDH, PFK, PKm and SOD-2. No significant differences between the HT and NC were detected by independent-samples *t*-test with Bonferroni correction for multiple comparisons (*P*>0.05) in relative changes of mRNA abundance. There were no significant differences between groups for heart rate and blood lactate during the treadmill test. The outcomes indicated that this hypoxia training program did not cause a significant variation in basal level expression of the selected mRNAs in Thoroughbreds as compared with normoxic training.

## Abstract (precis)

**Summary:** This was one of the first studies that examined transcriptional and physiological responses of Thoroughbred racehorses to six weeks moderate intensity treadmill training in a hypoxia chamber with 15% oxygen.

## INTRODUCTION

The quest for more effective and efficient training methods that result in optimal performance outcomes is a continuing area of interest in both human and equine fields, and in accordance with this, hypoxic training methods have been the focus of considerable scientific investigations on human athletes for several decades. However, to date only a small number of reports is available in the literature that have examined the physiological and transcriptional responses to hypoxia training in horses (Greene and Wickler, 2000; Nagahisa et al., 2016; Wickler and Anderson, 2000).

Traditionally, hypoxia training requires taking athletes to altitude where barometric pressure and oxygen partial pressure are lower. More recently, purpose-built chambers have been employed that allow the oxygen concentration to be lowered to varying levels within the chamber but at normal barometric air pressure (i.e. normobaric hypoxia). This reduced oxygen concentration may result in a reduced availability of oxygen for the cells.

Acclimations to hypoxic conditions occur due to intermittent or sustained exposure. In human studies, short-term exposure to altitude has shown positive acclimation in skeletal muscle (Friedmann-Bette, 2008; Hoppeler et al., 2008; Lundby et al., 2009). At the molecular level, intermittent training in hypoxia in human athletes has shown to result in an up-regulation of a regulatory factor, hypoxia-inducible factor-1 alpha (HIF-1α) (Hoppeler et al., 2008; Zoll et al., 2006). As a consequence of this up-regulation of HIF-1α, the level of mRNAs for myoglobin, vascular endothelial growth factor (VEGF), and glycolytic enzymes such as phosphofructokinase (PFK), together with mitochondrial and capillary densities, increased in a hypoxia-dependent manner (Zoll et al., 2006). However, sustained exposure to severe hypoxia has shown detrimental effects on skeletal muscle function with decreases in muscle oxidative capacity and loss of muscle mass (Hoppeler and Vogt, 2001). In addition to the potential benefits in endurance performance from hypoxia training, research has also suggested benefits to anaerobic exercise performance, via improvements in muscle buffering capacity (Gore et al., 2001) and glycolytic enzyme activity (Abe et al., 2015).

The application of the concept of hypoxic training to the Thoroughbred racehorse is new to the equine industry. To our knowledge there has been only limited research on this topic to date. The hypoxic training method used in horses partially simulates the ‘training high, living low’ approach used in human athletes. Despite some knowledge regarding the efficacy of hypoxia training for enhancing performance in the human athlete remaining debatable, the use of hypoxia training for equine athletes is increasing in popularity (Nagahisa et al., 2016; Rogers, 2013). The advent of these chambers necessitates further investigation of this training method before their use for training Thoroughbred horses can be advocated.

The aim of this study was to examine the effects of six weeks of normobaric hypoxic training when the fraction of oxygen in the inspired air (FiO_2_) was reduced from 0.21 to 0.15, simulating the partial pressure of oxygen at an altitude of approximately 3000 m, on mRNA levels at rest for a number of selected enzymes and regulatory factors in aerobic and glycolytic energy pathways in Thoroughbred horses.

In this study we investigated the transcriptional changes of selected genes which have been shown to be linked to aerobic performance: VEGF, HIF-1α, peroxisome proliferator-activated receptor gamma (PPARγ), peroxisome proliferator-activated receptor gamma co-activator 1-alpha (PGC-1α), cytochrome c oxidase subunit I (COX1), and cytochrome c oxidase subunit IV (COX4) (Eivers et al., 2010; Nagahisa et al., 2016; Zoll et al., 2006); glucose metabolism: lactate dehydrogenase (LDH), PFK, adenylate kinase (AK3), and pyruvate kinase muscle (PKm) (Abe et al., 2015; Greene and Wickler, 2000; Lundby et al., 2009); and oxidative stress: super oxide dismutase 2 (SOD-2) (Chung et al., 2005), in an endeavour to provide more insight into the potential impact of the hypoxic stimuli on Thoroughbred horses.

## RESULTS

**Fig. 1.**
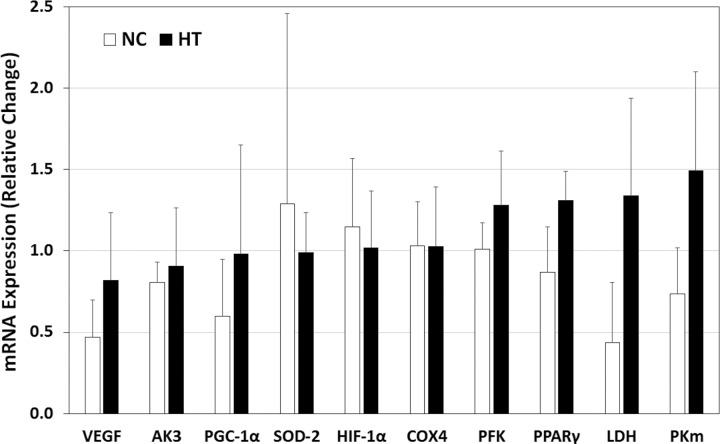
**Relative changes (fold change with reference to the pre-training value) in mRNA expression at rest as determined by the 2^−ΔΔCt^ method.** Data presented are group means with standard deviation. NC, normoxic control; HT, hypoxic training.

The general linear model with repeated measures (GLMRM) analysis on the ΔCt of mRNAs found no significant main effect of ‘training’ and interaction of ‘training by group’ for most mRNAs, except that the VEGF showed a significant training effect (*F*=6.808, *P*=0.040) and post hoc analysis indicated a significant increase post training in the normoxic control (NC) group; and PPARγ showed a significant interaction (*F*=6.609, *P*=0.042), and the post hoc analysis indicated that the NC had a trend of increase and the hypoxic training (HT) had a trend of decrease (Table 1). The independent samples *t*-tests demonstrated greater changes in mRNA expression in the HT group, in relative terms from pre to post training (2^−ΔΔCt^), as compared to the NC group in most measured mRNAs, with the non-corrected *P* values of 0.038 and 0.043 for between-groups comparisons for PPARγ and LDH, respectively. However, when the *P* values were corrected for multiple *t*-tests using Bonferroni correction, no significant differences were detected (*P*>0.05) (Fig. 1). One sample of COX1 was lost during analysis, therefore the mRNA COX1 results are not shown in Table 1 and Fig. 1.

**Table 1.**
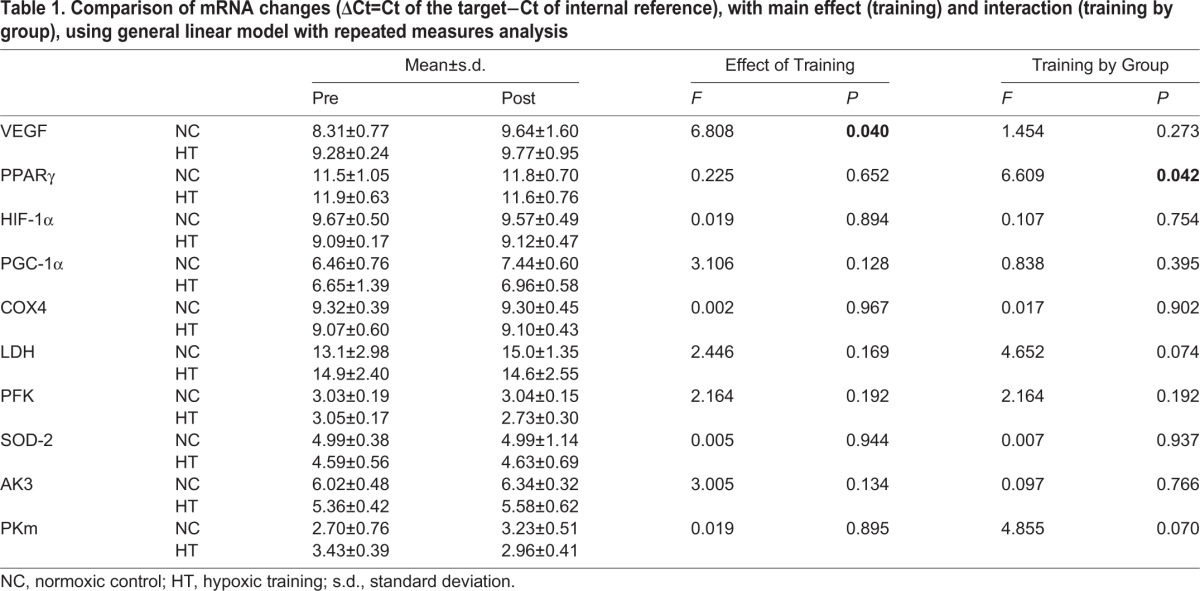
**Comparison of mRNA changes (ΔCt=Ct of the target−Ct of internal reference), with main effect (training) and interaction (training by group), using general linear model with repeated measures analysis**

All horses completed the test at treadmill speeds of 14, 21 and 28 km h^−1^ in both the pre and post training tests. For blood lactate changes, the results of GLMRM analysis showed a significant main effect of ‘training’ (*F*=37.093, *P*=0.001) and ‘speed’ (*F*=24.415, *P*=0.003), and significant interaction of ‘training by speed’ (*F*=11.070, *P*=0.015). Post hoc analysis with Bonferroni adjustment found that there were significant reductions from pre to post training in lactate at 14, 21 and 28 km h^−1^ (*P*=0.017, *P*=0.011 and *P*=0.003, respectively), but there were no significant differences (*P*>0.05) between the two groups at these speeds (Table 2).

**Table 2.**
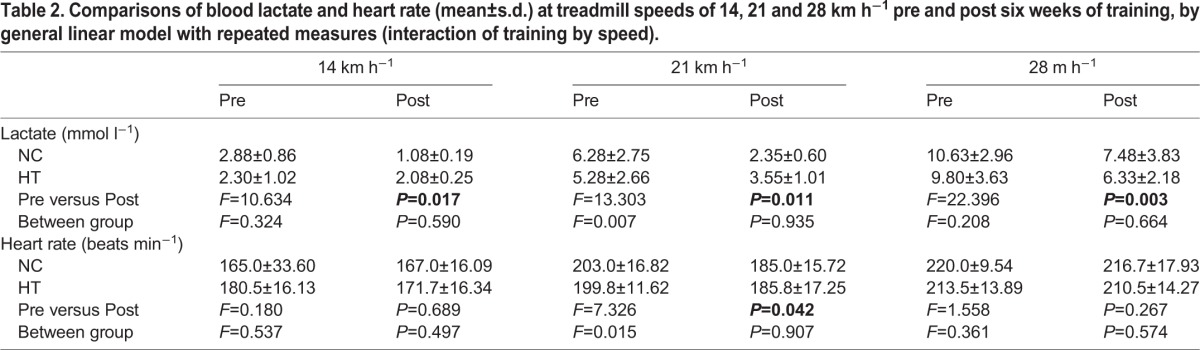
**Comparisons of blood lactate and heart rate (mean±s.d.) at treadmill speeds of 14, 21 and 28 km h^−1^ pre and post six weeks of training, by general linear model with repeated measures (interaction of training by speed).**

For the heart rate, there was a missing value in the pre training test of the NC group. Therefore the GLMRM was based on three horses for that group. There was a near significant main effect of training (*F*=6.369, *P*=0.053) with a trend of decrease in heart rate at higher speeds after training in both groups; and a significant main effect of speed (*F*=106.281, *P*=0.000) with higher heart rate at higher speeds (Table 2). However, there were no significant effects or interactions in all other analyses, except that at 21 km h^−1^ the heart rate was lower in the post training test (*P*=0.042).

## DISCUSSION

The results of this study showed that there were generally no significant differences between the NC and HT groups after six weeks of training for most of the investigated mRNAs, as well as the blood lactate and heart rate responses to an incremental treadmill test. However, trends can be seen (Fig. 1) in the changes in expression of mRNA at rest from pre to post training between the two groups. Although mRNA expression alone does not provide direct evidence to support variations in the abundance of proteins, the changes show a potential for possible impacts of hypoxia training on the glycolytic pathway (e.g. PFK, LDH and PKm) and aerobic metabolic system (e.g. VEGF, PGC-1α and PPARγ).

As this was one of the first training studies on Thoroughbred racehorses conducted in the hypoxic chamber, a positive outcome that can be drawn from the results was that the six weeks of training at the intensity utilized in a moderate hypoxia environment was well tolerated by the horses. The hypoxic stimulus used in this study, however, appeared to be insufficient to cause additional changes in the physiological responses to that of normal training as the decreased lactate and heart rates at submaximal workloads in the performance test were not significantly different between the two groups. A limitation of the research was the relatively small number of horses available in each group that would affect the statistical power. Furthermore, there is very little literature in the equine field in this area of research. While comparisons may be made with research in human athletes and rodents, caution should be exercised in interpretation of the results due to the differences in physiology and the greater muscle mass of the horses.

The lack of significant differences between the HT and NC groups for the physiological responses to the incremental treadmill test in the present study are in support of Holliss et al. (2013) who studied the effects of intermittent hypoxia (FiO_2_=0.145) and normoxia training in humans on muscle energetics, and reported that following the two training protocols there were essentially no differences in muscle metabolic responses to an incremental test (Holliss et al., 2013). Wickler and Anderson (2000) also reported no significant change in peak lactate values following altitude acclimatization in horses. In reviews by Pinilla (2014) and Hoppeler et al. (2008) it was reported that within the studies they reviewed there were no changes in maximal lactate concentrations with altitude training. In contrast, studies on rowers and runners showed lower lactate levels in post training tests (Bailey et al., 1998; Jensen et al., 1993). In the present study both NC and HT showed a significantly reduced blood lactate after training, indicating the effects of the training on muscle metabolism; however, the hypoxia training employed in this study did not result in a significant difference between the two groups in the physiological responses to the incremental submaximal test. Whether the blood lactate and heart rate measurements are sensitive indicators of the changes resulting from hypoxia training need to be further examined, with consideration of the mixed results from the literature.

Acute and chronic endurance exercise has been shown to affect oxidative capacity and metabolic efficiency of skeletal muscle in studies on humans (Pilegaard et al., 2003). The processes contributing to these changes have been considered to be associated with the cumulative effects of transient changes in gene expression (Egan et al., 2013; Pilegaard et al., 2003; Williams and Neufer, 1996), and subsequently an increase in mitochondrial protein content (Little et al., 2011; Perry et al., 2010). The transient changes of mRNA in response to a single training session often return to normal level within 24 h (Little et al., 2011; Perry et al., 2010; Pilegaard et al., 2003). Therefore, variations in mRNA level at rest could be an indicator of the cumulative effect of training or acclimation. For example, Lui et al. (2015) investigated the effects of 6-8 weeks acclimation to hypobaric hypoxia (4300 m) in deer mice, and compared the VO_2_max, muscle capillary density, and expression of genes involved in angiogenesis (including VEGFA) and energy metabolism (including PPARγ) between highlanders (captured at altitude 4350 m) and lowlanders (captured at altitude 430 m) (Lui et al., 2015). The authors reported that the phenotype of the highlanders, including greater VO_2_max, capillarity, oxidative fibre density and activities of oxidative enzymes, and lower LDH activity in the gastrocnemius muscle, was associated with higher mRNA and protein abundance of PPARγ. However, the transcript abundance of VEGFA was lower in highlanders, and hypoxia acclimation reduced the expression of numerous genes that regulate angiogenesis and energy metabolism. Scott et al. (2015) compared the aerobic capacity and oxidative enzyme activities in the skeletal muscle of highland with lowland deer mice, and reported that the expressions of the regulators of mitochondrial biogenesis, PGC-1α and mitochondrial transcription factor A (TFAM), were higher in the highland than the lowland deer mice (Scott et al., 2015). The evolutionary adaptations have occurred also in humans who have resided for many years at high altitude such as the South American Andes, the Tibetan Plateau and the Semien Plateau of Ethiopia (Bigham and Lee, 2014). The high altitude residents show elevated haemoglobin concentration, arterial oxygen saturation, pulmonary arterial pressure, and nitric oxide (regulating blood flow and vascular resistance) as compared with low altitude inhabitants. In addition to these physiological adaptations, the HIF pathway and a number of hypoxia-related genes have been identified that show genetic characteristics in these populations. For example, the prolyl hydroxylase domain protein 2 (PHD2) that controls erythropoietin via HIF-α is a common candidate gene in adaptation to altitude in Andeans and Tibetans. Several models have been identified with various combinations of the functional strengths of PHD2 and HIF2A in relation to the adaptations to high altitude (Bigham and Lee, 2014). In the present study, the transcriptional expression of HIF-1α did not show a significant change in response to the hypoxia intervention. It would be interesting to examine whether the above mentioned models are applicable to horses in future research.

In the equine field, Eivers et al. (2010) compared gene expression in skeletal muscle of Thoroughbred horses that were untrained with those that completed 10 months of training (Eivers et al., 2010). They reported that the transcriptional expression of the trained group for PGC-1α was 8% higher (non-significant), COX411 gene was 28% higher (*P*=0.02) and COX412 was 12% higher (non-significant) than the untrained group. In the present study there was only a small increase in COX4 (non-significant) with no difference between the NC and HT groups, while for the PGC-1α the HT showed a greater magnitude of change than the NC (non-significant). It has been hypothesized that training in a hypoxic environment would impose an additional stimulus and potentially lead to an improved performance. The physiological and cellular responses to hypoxic training that occur due to repeated or chronic exposure have been documented in human studies (Hoppeler et al., 2008; Vogt et al., 2001; Zoll et al., 2006), however with varied results. For example, Vogt et al. (2001) investigated the effects of six weeks training in normoxia and hypoxia (simulated altitude of 3850 m), at low and high intensity workloads. They reported that HIF-1α mRNA and mitochondrial density increased significantly after training under hypoxic conditions with both low and high intensity training. There was also a significant increase in VEGF, but only for the hypoxic high intensity training group (Vogt et al., 2001). Faiss et al. (2013) investigated the effects of four weeks repeated sprint cycling in either hypoxic (FiO_2_=0.146) or normoxic conditions in trained cyclists. They reported an increased number of sprint to exhaustion, together with increased mRNA level of HIF-1α, carbonic anhydrase III and monocarboxylate transporter-4, but decreased TFAM, PGC-1α and moocarboxylate transporter-1, in the hypoxic training group compared to the normoxic group, following a cycle performance test (Faiss et al., 2013). Ponsot et al. (2006) reported that the addition of two training sessions per week under hypoxic conditions (FiO_2_=0.145) to the normal training over a six-week period had meliorated the mitochondrial function and improved VO_2_max, VO_2_ at VT2 and time to exhaustion in endurance-trained athletes (Ponsot et al., 2006). Zoll et al. (2006) used the same protocol as Ponsot et al. (2006) and reported significant increases in mRNA levels of PGC-1α, PFK, COX1 and COX4, but no significant change in VEGF after training (Zoll et al., 2006). Robach et al. (2014) also compared the effects of six weeks training in normoxia or hypoxia (FiO_2_=0.15) on performance in an incremental cycling test in moderately trained men, however reported no significant differences between hypoxia and normoxia training in VO_2_max and skeletal muscle respiratory capacity and activity of COX (Robach et al., 2014).

The discrepancies as reported in the literature in relation to the expression level and type of genes may well be due to the protocols and the type of training used in the studies, therefore making comparative analysis complex. As the changes occurring at the transcriptional level do not always correlate with the translational outcomes, and as protein levels for the respective mRNAs were not measured in the present study, one can only make speculations about the potential impacts.

In summary, normobaric hypoxia training has recently been utilized as new training method for racehorses; however its efficacy and biological mechanisms of the acclimation to hypoxia training have not be thoroughly examined in the equine field. This study has shown that intermittent hypoxia training for six weeks may have a potential to cause additional changes in the selected gene transcription of the mitochondrial and glycolytic pathways of Thoroughbred horses, as compared with normoxia training, however further investigations with a larger sample size are required to determine optimal training programs, e.g. the level of oxygen, duration and frequency, for individual horses; and to understand the biological mechanisms of the acclimation.

## MATERIALS AND METHODS

Eight Thoroughbred horses, in the age range of two to five years, were used in the study. Horses were selected from a trainer's normal cohort in training, based on their performance history, best racing distance and time in training. Horses were randomly divided into two groups of four: normoxic control (NC), and hypoxic training (HT). All horses were trained using a high-speed treadmill (G. G. Engineering, Australia) and remained under the control of the trainer during the study. The horses were fed with a commercial concentrate (Hygain, Australia) and lucerne to meet equine nutritional guidelines.

The training program for both groups was the same. The program included alternate slow days and training days, with resting on Sunday during each week, for six weeks. On the slow days the horses exercised on the treadmill for 2 min walking at 6 km h^−1^, 5 min trotting at 12 km h^−1^, and 5 min cantering at 18 km h^−1^ at 4° elevation during the first two weeks; and 1 min walking at 6 km h^−1^, 8 min trotting at 12 km h^−1^, and 5 min cantering at 18 km h^−1^ at 4° elevation during the remaining four weeks. On the training days, the HT group trained within a hypoxic chamber at FiO_2_ 0.15, while the NC group trained under normoxic conditions. All horses progressed from low intensity warm-up of 2 min walk at 4-6 km h^−1^, 5 min trot at 12 km h^−1^ plus 3 min canter at 18 km h^−1^ at 4° elevation, followed by a progression from 2 to 3 bouts of 1 min gallops at 32 km h^−1^ at 6° elevation during the first two weeks; to 2×1 min gallops at 36 km h^−1^ at 4° elevation for week 3; then to 2×1 min gallops at 36 km h^−1^ at 6° elevation for week 4; then on two training days during weeks 5 and 6 they completed 3×1 min gallops at 36 km h^−1^ at 6° elevation; with 5 min recovery of walking and trotting between gallops in all weeks. This level of intensity in treadmill training is commonly utilized by trainers to prepare horses for development of a base level of fitness required prior to fast gallop work.

The hypoxia chamber is connected to a gas filtering system enabling oxygen to be extracted from the air, thus maintaining the FiO_2_ at 0.15. The rest of the air was nitrogen. The chamber was constructed by Pulford Air and Gas Company (Sydney, Australia).

### Treadmill testing

Prior to and on completion of the six weeks of training each horse completed an incremental performance test on the treadmill to determine the effects of training by examining physiological changes, including heart rate and venous blood lactate measured at each work load. The treadmill test protocol consisted of a warm-up involving walking for 1 min at zero grade, with grade then increased to 4° and the horse was trotted for 5 min. The incremental test was commenced following the trotting phase, with the grade increased to 6° and speed increased to 14 km h^−1^. The test consisted of 2 min at each of the speeds of 14, 21 and 28 km h^−1^. A Polar heart rate monitor (s810i) was used to continuously record heart rate during the incremental treadmill test.

### Blood sampling

Venous blood samples were collected for analyses of lactic acid concentration via a catheter inserted into the jugular vein. Catheter flow was maintained by occasional irrigation with sterile heparinised saline (4 IU ml^−1^). Samples were collected during the last 15 s of each workload. The samples were analyzed immediately for lactic acid using a Lactate Pro analyser (Arkray, Japan).

### Muscle biopsy

Muscle biopsies were taken one day prior to the pre-training test, and one day after the completion of the post-training test, for analysis of mRNA. Muscle biopsies were taken using the needle biopsy technique (Bergström, 1975). The biopsies were taken from the gluteus medium muscle at a depth of approximately 8 cm. Muscle samples were immediately placed into a sample tube containing RNAlater stabilization reagent (AMBION, Austin, TX, USA), then stored at −80°C before analysis.

### mRNA analysis

Total RNA was extracted from the muscle biopsy samples according to the manufacturer's speciﬁcations (TRIzol^®^ Plus RNA purification kit, Thermo Scientific, USA). The yield of RNA was determined using a NanoDrop 2000 spectrophotometer (Thermo Scientific, USA), and the integrity was evaluated using agarose gel electrophoresis stained with ethidium bromide.

Quantiﬁcation was performed with a two-step reaction process: reverse transcription (RT) and polymerase chain reaction (PCR). Each RT reaction consisted of 0.5 μg RNA, 2 μl of 5×PrimerScript Buffer, 0.5 μl of 50 μM oligo dT, 0.5 μl of random 100 μM 6mers and 0.5 μl of PrimerScript RT Enzyme Mix I (TaKaRa, Japan), in a total volume of 10 μl. Reactions were performed in a GeneAmp^®^ PCR System 9700 (Applied Biosystems, USA) for 15 min at 37°C, followed by heat inactivation of RT for 5 s at 85°C. The 10 μl RT reaction mix was then diluted by adding 90 μl nuclease-free water and stored at −20°C.

Real-time PCR (qPCR) was performed using LightCycler^®^ 480 II Real-time PCR Instrument (Roche, Switzerland) with 10 μl PCR reaction mixture that included 1 μl of cDNA, 5 μl of 2× LightCycler^®^ 480 SYBR Green I Master (Roche, Switzerland), 0.2 μl of 10 μM forward primer, 0.2 μl of 10 μM reverse primer, and 3.6 μl of nuclease-free water. Reactions were incubated in a 384-well optical plate (Roche, Switzerland) at 95°C for 10 min, followed by 40 cycles of 95°C for 10 s, 60°C for 30 s. Each sample was run in triplicate for analysis. At the end of the PCR cycles, melting curve analysis was performed to validate the speciﬁc generation of the expected PCR product. The primer sequences were designed in the laboratory and synthesized by Generay Biotech (Generay, China) based on the mRNA sequences obtained from the NCBI database (listed in Table 3). The levels of expression for the mRNAs (cycle threshold, Ct) were normalized to the internal reference glyceraldehyde 3-phosphate dehydrogenase (GAPDH) to obtain ΔCt (Ct of the target – Ct of internal reference) (Dundas and Ling, 2012; Nagahisa et al., 2016; Tariq et al., 2016). The housekeeping gene GAPDH appeared to be stable in this study, with the coefficient of variance of 4.9%. The differences in ΔCt between pre and post training values (ΔΔCt) were used to calculate the relative changes in gene expression using the 2^−ΔΔCt^ method (folds of change with reference to the pre-training value) (Livak and Schmittgen, 2001).

**Table 3.**
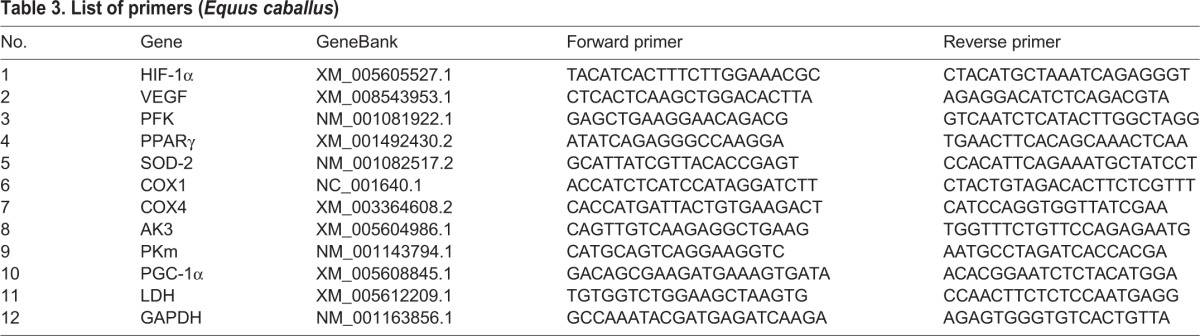
**List of primers (*Equus caballus*)**

### Statistical analysis

An independent samples *t*-test (two-tailed) was used to determine differences between the NC and HT groups for the relative changes pre to post training, as calculated from the 2^−ΔΔCt^ method, in mRNA expression of VEGF, PPARγ, HIF-1α, PGC-1α, COX1, COX4, AK3, LDH, PFK, PKm, and SOD-2, after six weeks of training. An alpha value of ≤0.05 was considered significant. Bonferroni correction was applied to the *P* values of multiple *t*-tests for a control of the risk of family-wise type 1 error (McDonald, 2009).

For the heart rate and blood lactate, the general linear model with repeated measures (GLMRM, IBM SPSS version 22) was used to determine the main effects of training (pre versus post) and group (NC versus HT), and the interaction between these two factors, for the ΔCt of mRNAs; as well as the main effects of treadmill speed (14, 21 and 28 km h^−1^) and group (NC versus HT), and the interactions between these factors. An alpha value of ≤0.05 was considered significant. If a significant effect or interaction was identified, post hoc analysis with Bonferroni adjustment was performed to determine where the significant difference existed.

The experimental protocol was approved by the Animal Care and Ethics Committee, Southern Cross University, Australia (approval number: ARA 14/11).
